# Receipt of core antenatal care components and associated factors in Ethiopia: a multilevel analysis

**DOI:** 10.3389/fgwh.2024.1169347

**Published:** 2024-02-21

**Authors:** Anagaw Derseh Mebratie

**Affiliations:** School of Public Health, Addis Ababa University, Addis Ababa, Ethiopia

**Keywords:** prenatal care components, pregnancy complications, nutritional counseling, health inequality, quality, Ethiopia

## Abstract

**Background:**

Despite recent promising progress, maternal morbidity and mortality are still unacceptably high in Ethiopia. This is partly attributed to the lack of quality health services. Pregnant women may not receive adequate services that are essential to protect the health of women and their unborn children. This study aimed to examine the extent of receiving prenatal care components and associated factors in Ethiopia. It also assessed prenatal service use inequality between urban and rural residents.

**Methods:**

The analysis was carried out using the 2016 Ethiopian Demographic and Health Survey (EDHS), which is nationally representative survey data. A weighted sample of 4,772 women nested within 595 communities who had live births five years preceding the survey was included in the study. Necessary adjustments were made to account for the design of the survey, and sampling weights were used to adjust for nonproportional allocation of the sample to strata. Bivariate and multivariable multilevel ordered logit models were used to analyze factors associated with receiving comprehensive ANC contents. Statistically significant predictors were identified at *p* value ≤ 0.05.

**Results:**

Among those women who had at least one ANC visit, only 15% (95% CI: 13, 16) received six core elements of antenatal care. The proportion of mothers who had essential prenatal components in rural areas was less than 13 percentage points. Approximately 43% of women did not receive at least two doses of tetanus toxoid vaccines to protect them and newborn infants against this life-threatening disease. Moreover, the majority of them, particularly those in rural Ethiopia, were not informed about pregnancy danger signs. Mothers who had at least four ANC visits received more types of prenatal components compared to those who had fewer ANC visits. The multilevel regression analysis revealed that receiving adequate ANC content is positively associated with having more frequent ANC visits, attaining a higher education level, being a member of a household in the highest wealth quintile and residing in urban areas.

**Conclusion:**

The evidence implies that the quality of maternal health services needs to be improved. Health programs and interventions should also give priority to rural areas where the majority of Ethiopian women reside.

## Introduction

Ethiopia has attempted to expand the supply of essential health services by implementing a 20-year Health Sector Development Program (HSDP) in the past two decades. Accordingly, between 2000 and 2019, there was a 17-fold increase in the number of health posts from 1,023 to 17,162 and a tenfold growth (382–3,678) in the number of health centers. In the same period, the number of public hospitals in the country increased from 80 to 314 (1, 2). The government has also invested to improve the availability of medical workers by expanding medical colleges throughout the country. As part of the HSDP, the Federal Ministry of Health (FMoH) also introduced an innovative community health extension program to enhance the utilization of maternal and child health services.

To reduce demand side constraints, the country also introduced an exemption policy that guarantees access to maternal and child healthcare services free of charge from public facilities (3). These efforts have contributed to enhancing the utilization of health services. For instance, in 2005, the proportion of institutional delivery was only 5%. In 2019, institutional delivery reached 48%. Between 2005 and 2019, the use of antenatal care services increased from 28% to 74%, and postnatal care service use in the first two days after childbirth rose from 5% to 34% (4, 5). There was also positive progress in reducing infant, child and maternal mortality. The country met the fourth Millennium Development Goal three years ahead of the 2015 deadline by reducing under-five mortality from 205 deaths per 1,000 livebirths in 1990 to 64 deaths per 1,000 livebirths in 2013 (6). Despite such promising progress, a large burden of preventable morbidity exists, and maternal mortality is still unacceptably high. In 2017, the maternal mortality ratio (MMR) in Ethiopia was 401 per 100,000 live births, which was categorized under the high MMR group according to the World Health Organization (WHO) standards (7).

The major causes of maternal mortality in Ethiopia are pregnancy-related and preventable cases such as hemorrhage, obstructed or prolonged labor, hypertensive disorders and infection (8, 9). The key factors contributing to these pregnancy complications are identified to be limited use of modern health services, poor competency of health providers and lack of facilities for obstetric services. Inequality in access to services is also a problem that needs to be addressed. Ethiopia is a predominantly rural country, and approximately 80% of the population lives in rural areas. However, the majority of rural women have limited access to quality health services during pregnancy and delivery (10–12). Accordingly, they may not receive a package of core antenatal care (ANC) services when they visit health facilities.

Since 2022, Ethiopia has adopted the 2016 WHO adopted model of a minimum of eight ANC visits and recipients of services that are effective in protecting the health of pregnant women and their unborn children (13, 14). The prenatal care provision guideline of the FMoH indicates that ANC services provided need to include blood pressure screening, weight measurements, check for pallor, fetal heartbeat and lie, urine test for infection, syphilis, blood group, hemoglobin, and rhesus factor screening. Moreover, pregnant women should also receive iron/folic acid supplements, insecticide-treated bed nets, a minimum of two doses of tetanus toxoid vaccination, deworming, nutrition counseling, and birth preparedness plans (15, 16). However, there is a marked gap in the quality of ANC service provision, and a number of pregnant women do not receive adequate ANC contents according to the national guidelines (17).

Previous studies have mostly investigated sociodemographic determinants of the prevalence and frequency of ANC visits in Ethiopia (18–21). However, there are limited studies that have examined the extent of ANC components and factors contributing to disparities among different segments of the population (16, 22). Related studies have also been conducted using demographic health surveys (DHS) in low- and middle-income countries. However, they did not account for the complex sample design nature of DHS data in the analysis (23–25). Failing to consider complex survey designs could lead to inconsistent coefficient estimates, and the conclusions drawn could be misleading. Moreover, DHSs often intentionally oversample smaller domains among different strata, and inferences made based on the unweighted sample data may be biased (26–29).

This study, therefore, aimed to assess the extent of ANC components received and identify associated factors in Ethiopia using the 2016 Ethiopia Demographic Health Survey. It also examined inequalities in the degree of ANC content provided in urban and rural residents. To do so, it employed appropriate research methods to analyze hierarchical survey data.

## Methods and materials

### Study design and sampling procedure

This study used the 2016 Ethiopia Demographic Health Survey (EDHS), which is a nationally representative household-based survey. A community-based cross-sectional study design was used in order to collect data from the source population. The sampling frame for the survey was the 2007 Ethiopian Population and Housing Census. Sample households were selected from urban and rural areas using a two-stage cluster sampling design. In the first stage, all regions in the country were categorized into urban and rural areas. Then, 645 enumeration areas (EAs) were selected considering probability proportional to the household size of the clusters. In the selected EAs, a household listing operation was implemented. In the second stage, 28 households per cluster were selected using an equal probability systematic sampling technique from the list of households created. Accordingly, a total of 18,060 households were allocated in the selected 645 EAs. However, due to the nonresponse rate, the survey contains completed interviews for 16,650 households (5,232 urban and 11,418 rural). Within sample households, there are 15,683 completed interviews with women of reproductive age (15–49 years old) (30).

Participants of this study were women aged 15–49 years who had a live birth and made at least one antenatal care visit in the five years preceding the 2016 EDHS survey. Accordingly, a weighted sample of 4,772 women who had ANC visits was included in the analysis.

### Variables and measurement

The outcome variables are components of ANC services received among those women who had at least one ANC visit during their recent live birth. The WHO recommends a core set of ANC services that include the following items: blood pressure measurement, tetanus toxoid vaccination, blood and urine tests, iron tablet supplementation, body weight measurement and counseling about danger signs. Receiving these contents is essential for every pregnant woman to prevent adverse maternal and perinatal outcomes (13). The 2016 EDHS includes information on these core ANC items except body weight measurement. Accordingly, for multivariate analysis, the outcome was measured as the number of core ANC services received out of six contents. In addition to these critical contents, the descriptive analysis also determines whether the women received the nationally recommended ANC items such as intestinal parasite drugs, nutritional counseling and information regarding a birth preparedness plan.

The regression analysis controlled three groups of factors to identify predictors of the outcome. First, it included the individual background characteristics of the mothers, such as age, religion, desire to have a child, number of ANC services received, marital status and education level. Second, the analysis also controlled for the wealth index of the households. Finally, it also included distance to health facilities and type of residence as proxies for community characteristics. Operational definitions of dependent and independent variables used in the bivariate and multivariate analysis are reported in Table 1.

**Table 1:** Operational definition of dependent and independent variables.

Variable	Description and categorization
Dependent variable	
ANC components	The number of core ANC components received during the last pregnancy. The core components include blood pressure measurement, tetanus toxoid vaccination, blood and urine tests, iron tablet supplementation and counseling about danger signs
Independent variable	
Age	Age in completed years: 15–19, 20–34, 35–49
Birth order	Birth order of the child: 1st birth, 2–3, 4–5, 6 or more
Wanted pregnancy	The mother desire status of last pregnancy: wanted by then, wanted latter, wanted no more
ANC visit	Number of ANC visit during the last pregenancy: 1, 2–3, 4 or more
Religion	Religious affiliation of the mother: Orthodox, Catholic, Protestant, Muslim, other religion
Marital status	Marital status of the woman: single, married, divorced, other marital status
Education status	Highest education level completed: no education at all, primary, secondary, higher education
Wealth index	The household relative wealth status: poorest, poorer, middle, rich, richest
Distance to the facility	Perception about problem related to distance to the facility: big problem, not a big problem
Residence	Place of residence: urban, rural

### Data analysis

The data were first extracted from the DHS Program website, and analysis was performed using STATA 16. The 2016 Ethiopian demographic health survey was collected using a two-stage cluster sampling design. To analyze data collected through such a complex sample design, it is important to know three types of information—the clustering variable or primary sampling unit, the stratification variable and the sampling weight variable (29, 31). Accordingly, this study was conducted after adjusting for these factors based on the information provided in the 2016 EDHS and using the STATA “svy” commands.

By taking the necessary adjustment for the sample design, the study described the background characteristics of the sample using frequencies and percentages. The distribution of receiving ANC components by urban/rural areas was reported using percentages and 95% confidence intervals (CIs). The weighted proportion of using core contents of prenatal care was estimated among women who had at least one ANC visit and those who made at least four visits. Moreover, multilevel regression models were fitted to identify predictors of the outcome. Classical regression models, such as logit and ordinary least squares, require independence of observations, and employing such models to analyze complex survey designs is not appropriate. In a hierarchical survey such as the EDHS, individuals within a cluster may exhibit similar characteristics, and observations with clusters may not be independent from each other. On the other hand, multilevel models account for cluster-level random effects and allow the dependence of sample individuals within a cluster (32).

Most of the existing studies [e.g. (23, 24, 33),] examined factors associated with receiving components of ANC using binary logit models, whereas this study employed bivariate and multivariable multilevel ordered logit model. The advantage of using ordered logits over binary logits is that the first ones measure the outcome on an ordinal scale. Hence, the models account for even small variations in the outcome among the study individuals, and the results are more informative. In the case of creating binary outcome variables, the precision of estimates decreases since information is lost in the process of combining categories of the outcome (34, 35). In this study, the outcome variable is measured on an ordinal scale and indicates the number of core ANC contents received by mothers included in the study.

As a robustness check, four alternative regressions were conducted after controlling for various covariates. The null model (Model 0) did not contain any explanatory factor, and its purpose was to assess the level of intracluster correlation across communities. Model 1 contained the background characteristics of sample women who had a live birth five years preceding the survey. Model 2 controlled for both individual and household characteristics. The final model (Model 3) incorporated individual-, household- and community-level factors.

Before conducting the multivariate analyses, multicollinearity tests were carried out to check the degree of relationship among the explanatory variables. It was found that the mean variance inflation factor (VIF) was 1.42, which was below the maximum tolerable value of 5, and predictors of the outcomes were not highly correlated (36). To assess differences in the extent of ANC content received across communities, intraclass correlation coefficients (ICCs) of the models were generated. The estimated ICC amounted to more than the 10% threshold, and it was important to control for community-level random effects to consistently estimate the coefficients of controlled covariates (37, 38). The relative proportion of community-level variance that was attributed to controlled factors in the consecutive models was assessed using proportional change in variance (PCV). Finally, crude odds ratios (CORs) and adjusted odds ratios (AORs) were estimated, and statistically significant predictors of ANC contents were identified at *p* values less than 0.05.

## Result

### Characteristics of the respondents

The study included a weighted sample of 4,772 women who received antennal care for their most recent birth in the five years preceding the 2016 EDHS. The sociodemographic characteristics of the study participants are reported in Table 2. The majority of women were 20–34 years old (73%), and they were married (91%). During the survey period, approximately 24% of the respondents had only one child, while 21% had six or more children. Most of the study participants were mainly Muslims (41%) and Orthodox Christian (39%). In terms of education status, approximately 33% of individuals attended primary education, while half of them did not attend any education at all. Most of the women reported that they gave birth to children mainly due to their desire to have a baby. Approximately 44% of the women stated that distance to the nearest facility was a major problem in accessing health services. Among the study participants, 3,317 (70%) resided in rural Ethiopia.

**Table 2:** Sociodemographic characteristics of women who received ANC in Ethiopia, 2016 (*N* = 4,772).

Variables	Category	Frequency	Percentage
Age (in years)	15–19	250	5.31
	20–34	3,432	72.84
	35–49	1,030	21.86
Birth order	1	1,151	24.43
	2–3	1,585	33.64
	4–5	1,003	21.29
	6 or more	973	20.65
Wanted pregnancy	Wanted then	3,765	79.90
	Wanted latter	695	14.75
	Wanted no more	252	5.35
ANC visits	1	342	7.26
	2–3	1,750	37.14
	4 or more	2,620	55.60
Religion	Orthodox	1,856	39.39
	Catholic	28	0.59
	Protestant	855	18.15
	Muslim	1,936	41.09
	Other	37	0.79
Marital status	Single	43	0.91
	Married	4,298	91.21
	Divorced	191	4.05
	Other	180	3.82
Educational status	No education	2,340	49.66
	Primary	1,543	32.75
	Secondary	521	11.06
	Higher	308	6.54
Wealth index	Poorest	1,068	22.67
	Poorer	761	16.15
	Middle	706	14.98
	Rich	671	14.24
	Richest	1,506	31.96
Distance to the facility	Big problem	2,088	44.31
	Not a big problem	2,624	55.69
Residence	Urban	1,395	29.61
	Rural	3,317	70.39

### Antenatal care visits

Among the weighted women who had live births in the five-year recall period, about 4.4% had only one ANC visit, while 26.5% had two or three visits. The survey indicates that 31.8% of mothers made at least four ANC visits. The characteristics of the mother who received at least 4 ANC visits are reported in Table 3. The results show considerable variations in having at least four ANC visits among different groups of women. For instance, 42% of women with first birth order made at least 4 prenatal care visits, while only 23% of those with 6 or more birth orders did the same. The chi-square test shows that there are statistically significant variations in the proportions of women who had a minimum four ANC visits based on the birth order of the recent child. Similarly, a higher percentage of mothers who attended higher education (73%) had repeated ANC visits than those with no education at all (24%). It is also found that a significantly higher percentage of women from households in the rich wealth status received at least four ANC services compared to those from poor households. In terms of place of residence, the proportions of women who had four or more ANC visits are 27% in rural areas and 63% in urban areas.

**Table 3:** Categorization of women who had at least four ANC visits during their recent pregnancy in Ethiopia, 2016.

Variables	Category	Frequency	Percentage	*p*-value
Age (in years)	15–19	104	30.7	0.0029
	20–34	1,781	33.7	
	35–49	530	27.0	
Birth order	1	602	42.0	0.0000
	2–3	808	35.4	
	4–5	524	29.9	
	6 or more	481	22.7	
Wanted pregnancy	Wanted then	1,853	33.3	0.0036
	Wanted latter	393	29.8	
	Wanted no more	168	24.2	
Religion	Orthodox	1,124	39.0	0.0000
	Catholic	20	27.8	
	Protestant	526	31.8	
	Muslim	726	25.7	
	Other	19	12.1	
Marital status	Single	34	60.7	0.0240
	Married	2,206	31.4	
	Divorced	81	34.6	
	Other	94	33.4	
Educational status	No education	1,156	24.1	0.0000
	Primary	828	38.5	
	Secondary	263	62.7	
	Higher	167	72.9	
Wealth index	Poorest	305	18.4	0.0000
	Poorer	419	25.3	
	Middle	447	28.1	
	Rich	517	36.2	
	Richest	728	57.4	
Distance to the facility	Big problem	1,108	25.1	0.0000
	Not a big problem	1,306	41.1	
Residence	Urban	608	62.7	0.0000
	Rural	1,805	27.3	

Chi-square (*χ*^2^) test was used to assess where there are significant variations in the proportions of at least four ANC visits among different categories.

### ANC contents received

**Figure 1:**
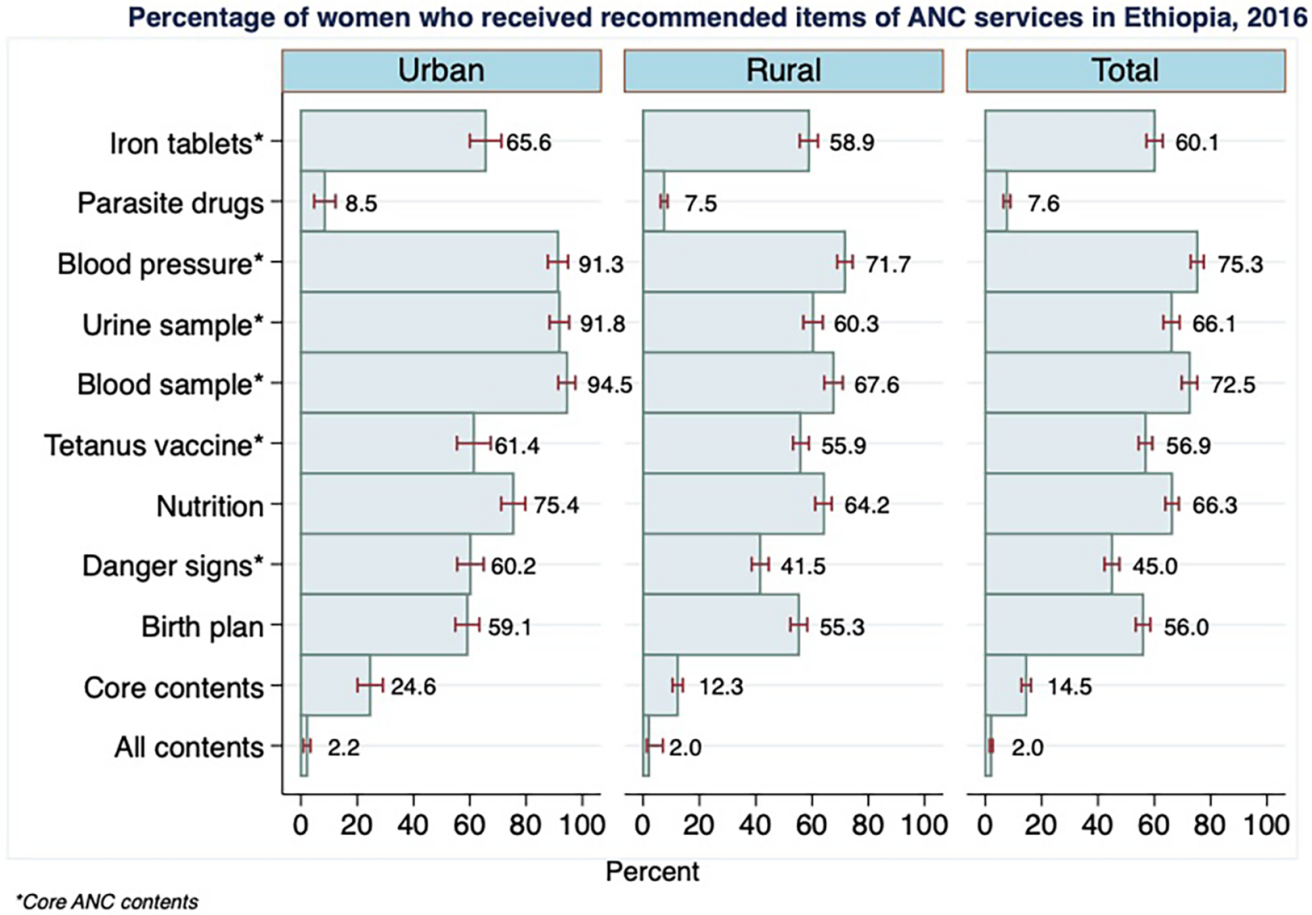
Percentage of women who received recommended items of ANC services in Ethiopia, 2016.

Among women included in the 2016 EDHS, 60% (95% CI: 57, 63) reported that they took iron tablets for their latest live birth (Figure 1). Relatively more women in urban areas (66%) than in rural areas (59%) received iron supplements. The blood levels of 91% of urban women and 72% of rural women were measured when they attended prenatal services. The proportions of women who had urine and blood tests amounted to 75% and 66%, respectively. Again, sample examinations were considerably higher for mothers in urban areas than for those in rural areas. For instance, the provision of blood sample test services was higher by 27 percentage points in urban areas. Approximately 57% of mothers took at least two tetanus toxoid injections. During their ANC visits, 66% of mothers received nutritional counseling, and 45% of them were informed about pregnancy danger signs.

The analysis revealed that only approximately 15% of women had a combination of six core components, which included iron tables, blood pressure measurements, urine and blood tests, tetanus injection and information on danger signs of pregnancy. The recipients of essential ANC components in rural areas were less by 13 percentage points than in urban areas. The proportion of women who received all nine contents included in the study was found to be only 2%.

**Figure 2:**
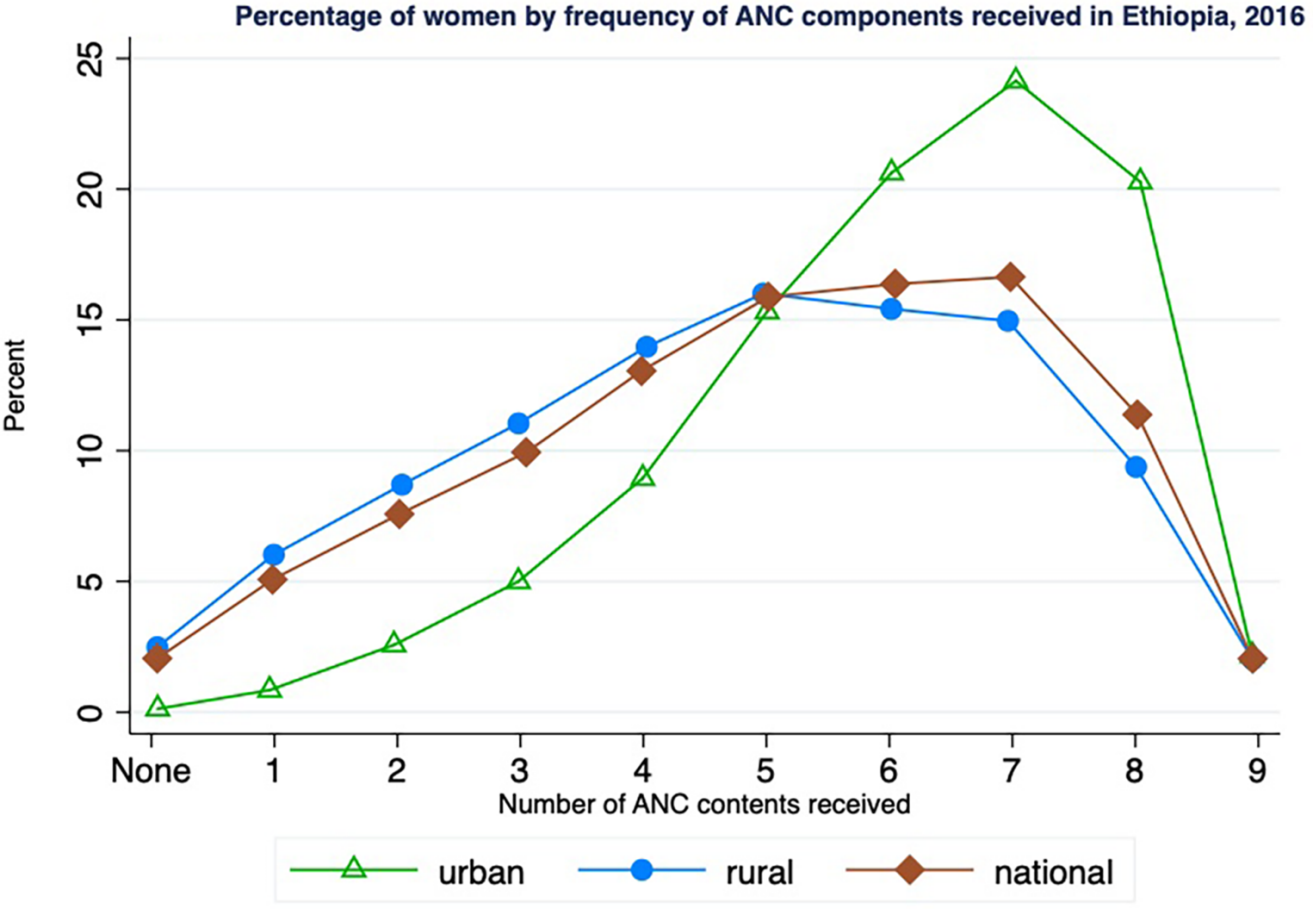
Percentage of women by frequency of ANC components received in Ethiopia, 2016.

Figure 2 indicates the proportion of women who received various degrees of ANC contents during their recent live births. Approximately 5% of women received only one ANC content, and 10% had three components. The share of women who reported having seven items was 17%. The proportion of individuals who had less than five contents was higher in rural areas than in urban areas, and the reverse holds true. Contrary to expectations, approximately 3% of rural women received none of the nine ANC items included in the analysis, although they reported using ANC services. On the other hand, almost all sample women in urban areas received at least one ANC component. The coverage of seven ANC contents was 24% in urban areas and 15% in rural areas.

The study also assessed prenatal packages among those women who availed at least 4 ANC visits during their recent pregnancy. As reported in Table 4, 67% of mothers having a minimum of four ANC visits received iron tablets, while only about 9% received parasite drugs. The proportion of women who had blood pressure measurements, urine tests, and blood tests were 81%, 76%, and 80%, respectively. The survey shows that 61% of women received tetanus vaccines and 73% received nutrition supplements. The percentage of women who received counseling about pregnancy danger signs and birth plans were 53.9% and 62.0%, respectively. Despite making frequent ANC visits, only a small proportion of women received all core ANC contents (21%) and all ANC contents (3%).

**Table 4:** Receiving prenatal contents among those women who had at 674 least four ANC visits in Ethiopia, 2016.

ANC content	Percent	95% CI
Iron tablets	66.5	[63.2, 69.6]
Parasite drugs	9.3	[7.6, 11.2]
Blood pleasure measurement	80.6	[77.8, 83.2]
Urine sample test	76.1	[73.0, 79.0]
Blood sample test	80.4	[77.4, 83.1
Tetanus vaccine	61.2	[57.9, 64.3]
Nutrition supplement	73.0	[70.3, 75.6]
Counseling about pregnancy danger signs	53.9	[50.4, 57.4]
Counseling about birth plan	62.0	[58.4, 65.2]
Receiving all core ANC contents	20.7	[18.1, 23.4]
Receiving all ANC contents	2.7	[1.9, 3.8]

### Factors associated with receiving higher items of ANC contents

The study examined factors affecting the extent of receiving ANC components using multilevel ordered log models. In these estimates, the outcome variable refers to the number of core ANC contents received by women. Table 5 provides crude odds ratios with 95% confidence intervals from the bivariate analysis. It is found that mothers who had four or more ANC visits had 5.6 times higher chance of receiving various types of contents compared to women who had only 1 ANC visit. On the contrary, women who wanted no more pregnancies had a lower chance of receiving all ANC contents compared to women who wanted their pregnancy at that time (COR = 0.68, 95% CI: 0.47, 0.97, *p*-value = 0.033). Individuals who attend post-secondary educational status had 3 times higher probability of receiving better prenatal services compared to women with no education (95% CI: 1.75, 5.20, *p*-value < 0.001). The results indicate a significant difference in the likelihood of receiving ANC contents based on place of residence. Women who resided in rural Ethiopia had 88% less chance of receiving more number of prenatal contents than those who resided in urban Ethiopia (95% CI: 0.16, 0.30, *p*-value < 0.001).

**Table 5:** Bivariate analysis of factors associated with receiving higher ANC components in Ethiopia, 2016.

Variables	Category	COR	95% CI	*p*-value
Age (in years)	15–19	1		
	20–34	1.27	[0.76, 2.12]	0.359
	35–49	1.06	[0.62, 1.79]	0.837
Birth order	1	1		
	2–3	0.90	[0.71, 1.14]	0.390
	4–5	0.73	[0.56, 0.94]	0.017
	6 or more	0.79	[0.60, 1.04]	0.093
Wanted pregnancy	Wanted then	1		
	Wanted latter	0.94	[0.74, 1.20]	0.617
	Wanted no more	0.68	[0.47, 0.97]	0.033
Number of ANC visit	1	1		
	2–3	2.83	[1.92, 4.16]	0.000
	4 or more	5.57	[3.79, 8.19]	0.000
Religion	Orthodox	1		
	Catholic	1.08	[0.33, 3.54]	0.905
	Protestant	1.06	[0.65, 1.73]	0.805
	Muslim	0.84	[0.58, 1.20]	0.325
	Other	0.55	[0.19, 1.54]	0.253
Marital status	Single	1		
	Married	0.92	[0.24, 3.45]	0.897
	Divorced	0.75	[0.18, 3.09]	0.692
	Other	0.60	[0.14, 2.56]	0.487
Educational status	No education	1		
	Primary	1.61	[1.29, 2.01]	0.000
	Secondary	2.63	[1.75, 3.97]	0.000
	Higher	3.02	[1.75, 5.20]	0.000
Wealth index	Poorest	1		
	Poorer	1.22	[0.89, 1.67]	0.212
	Middle	1.26	[0.90, 1.77]	0.177
	Rich	1.62	[1.14, 2.30]	0.007
	Richest	2.91	[1.81, 4.69]	0.000
Distance to the facility	Big problem	1		
	Not a big problem	1.14	[0.89, 1.47]	0.288
Residence	Urban	1		
	Rural	0.22	[0.16, 0.30]	0.000

In the multivariate analysis of Table 6, the estimate of the null model (Model 0) indicated that there were statistically significant variations in the odds of the ANC component receiving among clusters (variance = 1.68, *p* < 0.001). Similarly, the intraclass correlation coefficient (ICC) showed that approximately 34% of the total variance in receiving ANC contents was attributable to differences in the contextual factors across the communities (Table 2). The proportional change in variance (PCV) for models I, II and III were found to be 16%, 21% and 24%, respectively. Thus, in the final model, 24% of the community variance detected in the null model was explained by controlled individual-, household- and community-level factors.

**Table 6:** Multivariate analysis of factors associated with receiving higher components of ANC services in Ethiopia, 2016.

Variables (*N* = 4,772)	Model 0	Model 1	Model 2	Model 3
		AOR (95% CI)	AOR (95% CI)	AOR (95% CI)
Age (ref: 15–19)				
20–34		1.27 (0.71–2.28)	1.28 (0.71–2.30)	1.28 (0.71–2.30)
35–49		1.19 (0.61–2.32)	1.22 (0.62–2.39)	1.21 (0.62–2.38)
Birth order (ref: 1)				
2–3		0.98 (0.75–1.29)	0.96 (0.74–1.26)	0.96 (0.74–1.26)
4–5		0.86 (0.63–1.17)	0.85 (0.62–1.15)	0.85 (0.62–1.16)
6 or more		1.07 (0.75–1.54)	1.03 (0.72–1.47)	1.04 (0.72–1.48)
Wanted pregnancy (ref: Wanted then)				
Wanted latter		1.03 (0.81–1.32)	1.05 (0.83–1.34)	1.05 (0.82–1.33)
Wanted no more		0.81 (0.55–1.20)	0.82 (0.56–1.20)	0.81 (0.55–1.19)
Number of ANC visit (ref: 1)				
2–2		2.84* (1.92–4.19)	2.85* (1.92–4.21)	2.84* (1.92–4.20)
4 or more		5.47* (3.70–8.07)	5.41* (3.66–8.00)	5.38* (3.64–7.96)
Religion (ref: Orthodox)				
Catholic		1.02 (0.29–3.57)	1.03 (0.29–3.64)	1.05 (0.31–3.63)
Protestant		1.03 (0.65–1.62)	1.05 (0.67–1.65)	1.05 (0.67–1.65)
Muslim		0.89 (0.62–1.29)	0.92 (0.63–1.34)	0.92 (0.64–1.34)
Other		0.49 (0.20–1.21)	0.52 (0.21–1.28)	0.53 (0.22–1.29)
Marital status (ref: Single)				
Married		1.19 (0.33–4.31)	1.12 (0.31–4.06)	1.12 (0.31–4.07)
Divorced		1.02 (0.26–4.01)	0.99 (0.25–3.93)	0.99 (0.25–3.94)
Other		0.68 (0.17–2.74)	0.66 (0.16–2.69)	0.66 (0.16–2.70)
Educational status (ref: No education)				
Primary		1.56* (1.23–1.98)	1.50* (1.19–1.90)	1.50* (1.19–1.90)
Secondary		2.35* (1.52–3.64)	2.13* (1.39–3.26)	2.10* (1.36–3.23)
Higher		2.85* (1.66–4.89)	2.49* (1.41–4.39)	2.44* (1.37–4.33)
Wealth index (ref: Poorest)				
Poorer			1.08 (0.77–1.51)	1.08 (0.77–1.50)
Middle			1.08 (0.77–1.52)	1.08 (0.76–1.52)
Rich			1.26 (0.89–1.78)	1.25 (0.88–1.77)
Richest			2.09* (1.29–3.40)	1.97* (1.18–3.29)
Distance to the facility (ref: Big problem)				
Not a big problem				1.07 (0.84–1.37)
Residence (ref: Urban)				
Rural				0.58* (0.36–0.94)
Random effects				
Community level variance (SE)	1.68* (0.17)	1.42* (0.19)	1.32* (0.18)	1.28* (0.17)
ICC	*0.338*	0.302	*0.286*	*0.280*
PVC	*Ref*	0.155	*0.214*	*0.238*

Model 0—null model and no covariates controlled for; Model 1—Controlling for individual level factor; Model 2—Controlling for household level factors; and Model 3—Controlling for community level factors.

* Significant at *p* < 0.05.

The sociodemographic characteristics of the mothers, such as age, marital status and religion, were not found to be important predictors of ANC components. The results show that the number of ANC visits was positively associated with the content of prenatal services. Keeping other factors constant, the number of ANC contents received was 2.84 times greater for those who made two or three ANC visits than for those who made only one ANC (95% CI: 1.92, 4.20, *p*-value < 0.001). Similarly, the components of ANC services were 5.38 times higher for women who made three or more visits (95% CI: 3.64, 7.96, *p*-value < 0.001). The results were consistent across alternative model specifications (Table 5).

Education was also found to be an important predictor of receiving ANC contents in the study country. Those who attended primary education were 1.5 times more likely to obtain comprehensive services than those who did not attend formal education. The adjusted odds ratios for women with secondary education and those with higher education were 2.10 (95% CI: 1.36, 3.23, *p*-value = 0.001) and 2.44 (95% CI: 1.36, 3.23, *p*-value = 0.002), respectively. Perceived distance to the health facility did not significantly relate to the outcome of interest. Finally, those who lived in rural Ethiopia had less ANC services than those in urban areas (AOR = 0.58, 95% CI: 0.36, 0.94, *p*-value = 0.026).

## Discussion

In the past two decades, Ethiopia has made considerable progress in expanding access to maternal and child health services. This leads to noticeable progress in enhancing service utilization in the country. Accordingly, the proportion of women who had at least one antenatal care service increased from 28% in 2005 to 74% in 2019. The utilization of a minimum of 4 ANC visits also markedly improved from 12% to 43% (5). However, using the 2016 EDHS, this study indicates that the progress made in expanding access to prenatal services is not accompanied by the provision of adequate services required for a better pregnancy outcome. It was found that the share of women who had essential ANC components was limited in the country. Among women who had at least one ANC visit five years preceding the survey, 85% did not receive six items of core ANC services. Relatively more rural women (88%) did not receive the required services compared to urban women (75%). On the other hand, those women who made at least ANC visits received more types of prenatal components than those who had fewer visits. The evidence implies that many pregnant women, especially those in rural areas, were not protected from the risk of complications that could endanger the lives of both the mothers and their babies. In addition to demand-side constraints, a lack of quality services is a problem that causes inadequate use of prenatal contents in Ethiopia (11, 12).

The share of women having essential ANC service in the 2016 EDHS was relatively larger than a previous study conducted in Nigeria (5%) but smaller than studies in Nepal (23%) and Bangladesh (22%) (23–25). Evidence from the Performance Monitoring for Action 2020 Ethiopian data showed that 28% of pregnant women received adequate ANC content (16). This study considered adequate receiving of care if the pregnant women received at least 9 out of 12 items of ANC contents. However, in the current study, the outcome was defined as receiving six core ANC components during a recent live birth.

Measurement of blood pressure and examinations of urine and blood samples were found to be the most common ANC services provided in Ethiopia. This is consistent with evidence in Nigeria and Bangladesh, and this is because these are the very basic services provided during the first ANC visits (23, 25). Similar to a study conducted in rural Western Kenya, only less than half of mothers were informed about the danger signs of pregnancy during their ANC visits (39). Approximately 43% of women did not receive the minimum required doses of tetanus toxoid vaccine. This is not according to the Ethiopian ANC service provision guideline and the WHO recommendation of at least two tetanus toxoid injections during the pregnancy period to prevent maternal and neonatal mortality (40, 41).

The study overall indicates that, despite visiting health facilities to seek ANC, many women did not receive adequate services. This is mainly due to a significant health systems failure to deliver essential maternal health services. According to the 2016 Service Availability and Readiness Assessment (SARA) for Ethiopia, Folic acid supplementation were available only in less than 60% of the health facilities that provide antenatal health services. Similarly, iron supplementation and tetanus toxoid vaccination were not available in about one quarter of health facilities (42).

The estimate from multilevel regression analysis revealed that making more than one ANC visit contributed to receiving a higher number of ANC components. The WHO also recommends that pregnant women make at least four ANC visits to ensure the receipt of necessary prenatal services (13). When pregnant women visit health facilities more frequently, they obtain relevant information and medications that are life saving for both the mothers and their children. The contents of ANC services provided by health facilities also differ according to the pregnancy stages, and women who make only one or two visits may not receive adequate ANC content (15). Similar to this evidence, a study from Northern Ethiopia found that the number of antenatal care visits was significantly associated with the incidence of tetanus toxoid immunization (41). Related studies in Nigeria and Nepal also indicated that the frequency of ANC visits significantly predicted the contents of services being received (23, 24).

Education was also found to be an important confounder that determined the extent of ANC content received, which was consistent with previous studies in Ethiopia, Nigeria and Bangladesh (16, 23, 25). Educated women could be more aware of pregnancy-related complications and methods to prevent them. Hence, they could also have better healthcare-seeking behavior toward antenatal care services than those who were not educated.

The economic status of the households was also significantly related to the extent of ANC services received. Consistent with results in Nepal and South Ethiopia (24, 43), the poorest segment of the societies was less likely to receive more ANC services compared to the wealth group. This could be due to a lack of financial capacity to cover the direct medical and nonmedical costs required to receive health services. On the other hand, the rich could use more frequent and quality ANC even when service provision prices were expensive to the majority of the households.

Finally, the empirical analysis indicated that the location of residents significantly affected the utilization of antenatal services. In line with reports of studies from Nigeria, Nepal, and Ethiopia, rural residents were less likely to receive ANC components than urban residents (16, 23, 24). Even if access to health care services had expanded in various parts of Ethiopia, there was still urban rural disparity in obtaining access to quality services. Shortages of medical equipment and essential drugs were common problems in health facilities located in rural Ethiopia, where 80% of the population resides. Midwives working in rural public facilities struggled to save the lives of women due to a lack of essential medicines and devices, including thermometers, stethoscopes and weighing scales. Laboratory services were also limited due to scarcity of detergents and equipment (12, 44). Rural people also face transportation and financial constraints to access services available from far distances to their locations (16, 45). In addition, awareness and demand for health services were also poor in rural parts of the country (12, 46–48).

This study provides evidence on the quality of ANC service in Ethiopia by examining the extent to which women receive essential prenatal care components using a nationally representative household-based survey and robust statistical methods. However, the interpretation of the findings needs to be made considering the following limitations. In the study, components of ANC services received were assessed without considering the trimester at the first ANC visit. The 2016 EDHS does not include information on body weight measurement, which is one of the WHO recommended core components of ANC services (13). Moreover, the study is based on a survey that was conducted in 2016 and the results may not reflect recent situations. This is because, during the time of this study, there were no recent demographic and health surveys that contained relevant indicators on the contents of prenatal care received among women who had at least one ANC visit.

## Conclusion

The study revealed that the majority of women, especially those in rural Ethiopia, did not receive the essential ANC components required to ensure safe delivery. This calls for designing appropriate policies to expand coverage of adequate ANC service provision in the country. In this regard, it is primarily important to strengthen the health system and make essential maternal health services available in health facilities. To enhance awareness about pregnancy-related complications and preventive methods, creating educational opportunities for women could also be useful. This also encourages women to properly plan pregnancy and to seek maternal health care services. It is also important to expand quality health care services in rural Ethiopia, where there is low utilization of essential health services. Due to scattered settlement and low affordable capacity, there are currently limited private providers in rural Ethiopia. Hence, governmental and nongovernmental organizations need to exert efforts to improve access to essential maternal health services in rural areas. The health agendas of sustainable development goals (SDGs) are also less likely to be achieved unless efforts are being made to reduce maternal mortality and improve the health status of women in rural Ethiopia.

## Data Availability

Publicly available datasets were analyzed in this study. This data can be found here: The data used for this study were obtained from the Demographic and Health Surveys (DHS) Program and are accessible with approval from the DHS Program.
